# Cocoas and Chocolates

**Published:** 1894-09-01

**Authors:** 


### PRACTICAL DEPARTMENTS.

### COCOAS AND CHOCOLATES.

#### II.—Solubility, Digestibility, and Adulteration.

It has now been ascertained by experiment that the addition of an alkali in the small proportions necessary to render the cocoa more soluble is not only harmless, but, in a sense, beneficial to the consumer. It is beneficial in the facts that it makes cocoa drinking much more pleasant and facilitates its preparation for consumption by reducing the sediment and freeing it from lumpiness. It is to be regretted, however, that some manufacturers have started the cry that the addition of any alkaline matter is harmful, especially as they forget that the ash of the nut itself contains a large quantity of phosphate of potash. We can, therefore, dismiss this alkaline controversy with the remark that the amount of added matter required to secure the necessary degree of solubility is so small as to be quite harmless.

The profession and the public ought further to be made aware that prepared cocoas and chocolates are articles of commerce the manufacturers of which cannot be proceeded against for adulteration, provided the character of the article is mentioned on the label attached to each package. A recent case before the Court of Appeal, Jones v. Jones, is one in point. This appeal was first taken to Quarter Sessions against a decision of the justices sitting at Pontypridd, by which they convicted the appellant for having sold a certain article of food "not of the substance, nature, and quality of the article demanded by the purchaser." In other words it was argued that the article sold was not cocoa, as demanded by the purchaser, but an admixture of starch, sugar, and cocoa, to the prejudice of the consumer. A fine of 1s. and costs was inflicted, and against this decision an appeal was made, first at Quarter Sessions, where the Court was divided in opinion, and subsequently to the High Court. The purchaser was an inspector under the Sale of Food and Drugs Act, and sent the cocoa to the county analyst, who certified that the sample contained 30 parts of cocoa and 70 parts of starch and sugar mixed therewith. The manufacturers of this cocoa had sold it for thirty years, under the same name, "Pearl Cocoa," and the label contained these words: "Cocoa combined with other ingredients, the perfect purity and wholesomeness of which are guaranteed." The retail price of pure cocoa nibs was 1s. 5d. per pound, of pure soluble cocoa 2s. 8d. per pound, but "Pearl Cocoa" was sold at 8d. per pound. It was contended for the appellant that the label on the packet was a sufficient protection under the Act of Parliament, and had been accepted as such, but the defendant maintained that as the packet was delivered to him in white paper, with no notice of the label, it was a delivery which brought the appellant within the meaning of the section of the Act. The price charged showed that the admixture was not made fraudulently to increase the bulk, weight, or measure of the article, or to conceal its inferior quality. Mr. Justice Mathew and Mr. Justice Cave, sitting as a Divisional Court, held that the conviction was wrong, and that the article sold was a well-known article, manufactured by the same people and in the same way for more than thirty years, and always sold of the same quality. The label complied with the Sale of Food and Drugs Act, 1875, and in clear terms informed the purchaser that the article was "Pearl Cocoa," so giving sufficient notice; further, the suggestion that there was fraud was not sustained by the slightest evidence of intention to defraud on the part of anybody, and the ingredients of the admixture were admitted to be quite wholesome; the appeal was consequently allowed with costs. It follows that manufactured articles, where the ingredients are admittedly wholesome and calculated to improve the prepared food and to make it more palatable and readily available for preparation for the table, are an advantage to the consumer, and can in no sense be regarded as adulterants. As well might a baker who also sells cakes and biscuits be charged with adulteration. Indeed, we would go further, and state that, so far from blame, any manufacturer is entitled to credit for inventing a recipe which shall make such an important article of diet as cocoa undoubtedly is, more popular with increasing numbers of the population. Unfortunately cocoa is adulterated, as Dr. Hassall has shown in his book on adulteration, where he states that he found thirty-nine out of sixty-eight samples contained earthy colouring matter, such as reddle, Venetian red, and umber. It is to be hoped that no British manufacturer is responsible for adulterations of this kind.

We have shown, then, that cocoa itself contains a certain amount of alkaline matter, and that a small addition of further alkaline matter for the purpose of making it more soluble and palatable is permissible, and may even be desirable, because it brings this food into use by many persons who would otherwise be unable to take it.

